# Modification and functional adaptation of the MBF1 gene family in the lichenized fungus *Endocarpon pusillum* under environmental stress

**DOI:** 10.1038/s41598-017-16716-4

**Published:** 2017-11-27

**Authors:** Yanyan Wang, Xinli Wei, Jenpan Huang, Jiangchun Wei

**Affiliations:** 1 State Key Laboratory of Mycology, Institute of Microbiology, Chinese Academy of Sciences, Beijing, 10010 China; 2 Science & Education, The Field Museum, Chicago, IL 60605 USA; 3 University of Chinese Academy of Sciences, Beijing, 100049 China

## Abstract

The multiprotein-bridging factor 1 (MBF1) gene family is well known in archaea, non-lichenized fungi, plants, and animals, and contains stress tolerance-related genes. Here, we identified four unique *mbf1* genes in the lichenized fungi *Endocarpon* spp. A phylogenetic analysis based on protein sequences showed the translated MBF1 proteins of the newly isolated *mbf1* genes formed a monophyletic clade different from other lichen-forming fungi and Ascomycota groups in general, which may reflect the evolution of the biological functions of MBF1s. In contrast to the lack of function reported in yeast, we determined that lysine^114^ in the deduced *Endocarpon pusillum* MBF1 protein (EpMBF1) had a specific function that was triggered by environmental stress. Further, the *Endocarpon*-specific C-terminus of EpMBF1 was found to participate in stress tolerance. *Epmbf1* was induced by a number of abiotic stresses in *E. pusillum* and transgenic yeast, and its stress-resistant ability was stronger than that of the yeast *mbf1*. These findings highlight the evolution and function of EpMBF1 and provide new insights into the co-evolution hypothesis of MBF1 and TATA-box-binding proteins.

## Introduction

Multiprotein-bridging factor 1 (MBF1) is a transcriptional co-activator. MBF1 acts as a linker between TATA-box-binding proteins (TBPs) and sequence-specific transcription factors to activate transcription of downstream genes^1^. MBF1 contains an N-terminal domain of about 40-amino acids, a conservative helix-turn-helix (HTH) domain and a short C-terminus. The N- and C-termini sequences are divergent among different organisms, whereas the HTH domain, which contains four α-helices, is conserved^2^. The HTH domain is responsible for the functional activity of MBF1, and differences in the N- and C-termini do not affect the main activities of MBF1 proteins^3,4^.

In the yeast *Saccharomyces cerevisiae*, the TBP-binding site of MBF1 (yMBF1) is an aspartate residue (D) at position 112, which is located in the third α-helix, and the corresponding interacting site in yTBP is a glutamine residue (Q) at position 68^5^. A compensatory change analysis found that the yMBF1^D112K^ mutant [D^112^ changed to lysine (K)^112^] did not interact with yTBP in yeast^5^. This site (D^112^ in yMBF1) is conserved in this position in most fungal MBF1s^6^, while the D is replaced by glutamic acid (E) in plant and animal MBF1s. The corresponding binding site in the TBPs (position 68 of yTBP) is also highly conserved. Thus, it has been hypothesised that the interacting amino acids between MBF1 and TBP co-evolved^5^. Even the parasitic protozoan *Cryptosporidium parvum*, which has lost many essential genes from its genome, maintains this relationship between MBF1 and TBP^7^.

Gene knock-out strains (Δ*mbf1*) of different organisms were found to be viable although they displayed some functional faults^8^. The affected functions varied among different organisms, for example, histidine synthesis in yeast, development in plants, and hyphal morphogenesis, stress response, and virulence in filamentous fungi^8–13^. Nevertheless, *mbf1s* from *Arabidopsis*, invertebrates, and vertebrates were capable of complementing the yeast Δ*mbf1* mutant^14,15^. These findings suggested that the MBF1 domain for binding basic transcription factors was conserved, but whether the variable sequence determined the species-specific functions requires further investigation.

MBF1s have been reported in diverse eukaryotes and archaea, but are absent in eubacteria^12,14^. The known MBF1s, from Archaea, Fungi, Plantae, and Animalia have been classified into three groups: (I) MBF1 homologs in Archaea; (II) MBF1 homologs in Animalia, Protista, Plantae, and certain groups of Fungi; and (III) MBF1 homologs in Pezizomycotina, Fungi^13^. Most archaeal HTH-containing proteins have been obtained from bacteria through multiple horizontal gene transfer events^16^. The absence of MBF1 homologs in eubacteria suggests that MBF1 was present originally in the last common ancestor of archaea and eukaryotes^14^. Gene duplication events occurred only in the *Arabidopsis thaliana* genome^15^. A phylogenetic tree of archaea grouped MBF1s from hyper-thermophiles into a separate branch^14^. However, the evolutionary history of the MBF1 family is not clear. In this study, we identified additional homologs of MBF1 in lichens and we investigated whether changes in the amino acid sequence of MBF1 unique to the desert lichen *Endocarpon pusillum* Hedw. affect stress tolerance, which is an important hypothesized function of these proteins^13,17^. Like other lichens, *E. pusillum* is composed of a mycobiont (the lichenized fungus *E. pusillum*) and a photobiont (the green alga *Diplosphaera chodatii* Bialosuknia). The former can survive for seven months under desiccation stress combined with starvation stress, and for eight months under starvation stress alone, while the latter can survive only two months under desiccation stress^18^. Thus, it is intriguing to understand the functions of the *mbf1* gene of *E. pusillum* and determine if the MBF1 protein family undergoes environmental selection during the evolutionary process.

## Results

### Isolation of *mbf1* from *Endocarpon pusillum* and sequence analysis of MBF1

#### MBF1s in *Endocarpon* spp. and other lichenized fungi

The *mbf1* gene from the lichenized fungus *E. pusillum* (*Epmbf1*) was isolated and cloned. The genomic sequence of *Epmbf1* is 560 bp in length with a single 59-bp intron, and the full-length cDNA of *Epmbf1* (GeneBank: HM193140) is 501 bp encoding a protein of 166 amino acid residues. A BLASTP analysis of the deduced amino acid sequence revealed that EpMBF1 shared 46% and 41% identities with the MBF1 sequences of *Fusarium verticillioides* (GenBank: EWG40545.1) and *S. cerevisiae* (GenBank: BAA33217.1), respectively.

Based on the coding sequence of *Epmbf1*, three *mbf1s* were amplified from other *Endocarpon* species, i.e. *E. crystallinum*, *E. nigromarginatum*, and *E. sinense* (GenBank: KX364209, KX364211, and KX364210, respectively). To determine whether MBF1s from lichen-forming fungi are all unique, five species were chosen, *Cladonia metacorallifera* ^19^, *Cladonia macilenta* ^20^, *Gyalolechia flavorubescens* ^21^, *Umbilicaria muehlenbergii* ^22^, and *Lasallia pustulata* (GenBank assembly accession: GCA_900169345.1), which are all the lichen species up to now having genomic data in the NCBI. The corresponding deduced MBF1 sequences were annotated using a local BLASTP search tool.

The multiple sequences alignment (Supplementary Fig. S1) showed that there was an extra 14~15-amino acid fragment at the C-termini of the MBF1s in *Endocarpon* spp. when compared with other non-lichenized fungi. Except for the extra amino acids region at the C-terminus, the sequences of all the fungal MBF1s were similar. The amino acids in interaction loci were also different. As previously reported^5^, there was a D at the interaction loci in most non-lichenized fungi (indicated by an arrow in Supplementary Fig. S1). In the MBF1s of *Endocarpon* spp. there was a K at the corresponding position 114, and in other lichenized fungi, there was a K, threonine (T), or leucine (L) at the corresponding position, indicating the residues in this position were not conserved among lichenzied fungi.

#### Sequence analysis of MBF1

As reported in the previous study^13^, MBF1s from Archaea, Fungi, Animalia, and Planta were classified into three groups by neighbour joining (NJ) tree. A dataset containing lichenized fungal MBF1 sequences were analysed in this study by constructing a NJ tree and a Bayesian tree. The NJ tree (Supplementary Fig. S2) was constructed based on the aligned amino acid sequences of the MBF1s using the Poisson model (Tree base submission No.: 21188). In the NJ tree, *Endocarpon* spp. clustered together with other fungi of Pezizomycotina with a rather low bootstrap value (41%), suggesting an uncertain position of this group (Supplementary Fig. S2).

**Figure 1:**
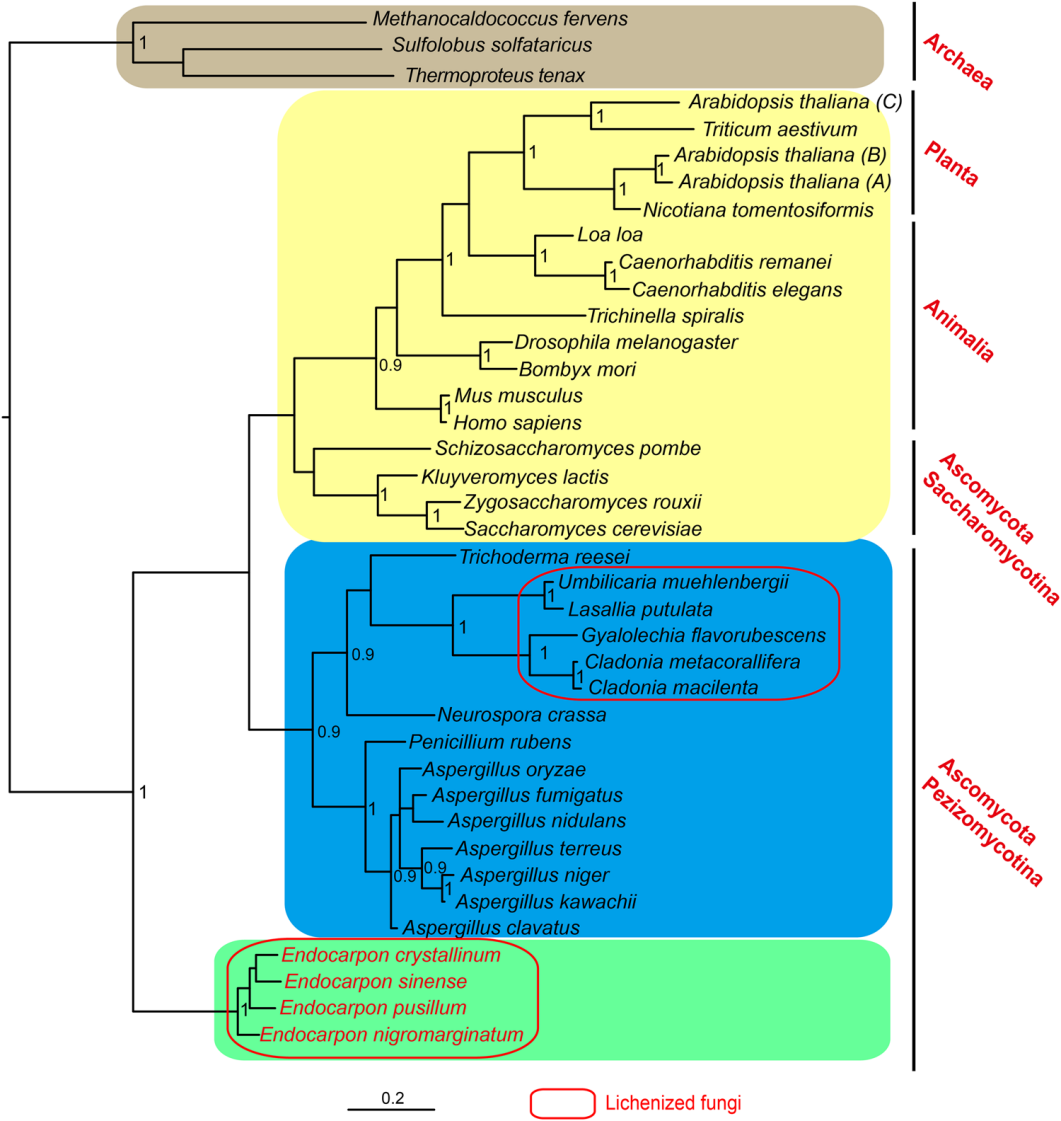
Bayesian phylogenetic tree based on the amino acid sequences of MBF1s. The Bayesian tree was constructed with MrBayes 3.2.6 on the CIPRES Science Gateway. The 39 MBF1 sequences represent Archaea, Fungi, Animalia, and Planta (see Supplementary Table S2). Different colours indicate the different groups. The numbers at each node represent posterior probability values. Numbers greater than 0.90 are shown above branches. Scale bar = 0.2 substitutions per site.

A clear topology structure was obtained from the Bayesian Markov chain Monte Carlo sequence analysis (Fig. 1), which was constructed based on the aligned amino acid sequences of the MBF1s using the best model of LG + G + I. The phylogenetic tree showed that the MBF1s clustered into four groups (Fig. 1): Group I contained the MBF1s from Archaea; Group II contained the MBF1s from the Animalia, Planta, and Saccharomycotina in Ascomycota; Group III contained the MBF1s from fungi of Pezizomycotina in Ascomycota; and Group IV contained the MBF1s from *Endocarpon* spp., belonging to Chaetothyriomycetidae, Eurotiomycetes, Pezizomycotina, Ascomycota. The first three groups were the same as the result of previous study^13^. The five lichen-forming fungal MBF1s from *C. metacorallifera*, *C. macilenta*, *G. flavorubescens*, *U. muehlenbergii*, and *L. pustulata* clustered into Group III and were different from those of the *Endocarpon* spp. (Group IV).

### Stress-resistance related functions of *Epmbf1*

#### *Epmbf1* is induced in *E. pusillum* cells under abiotic stress

**Figure 2:**
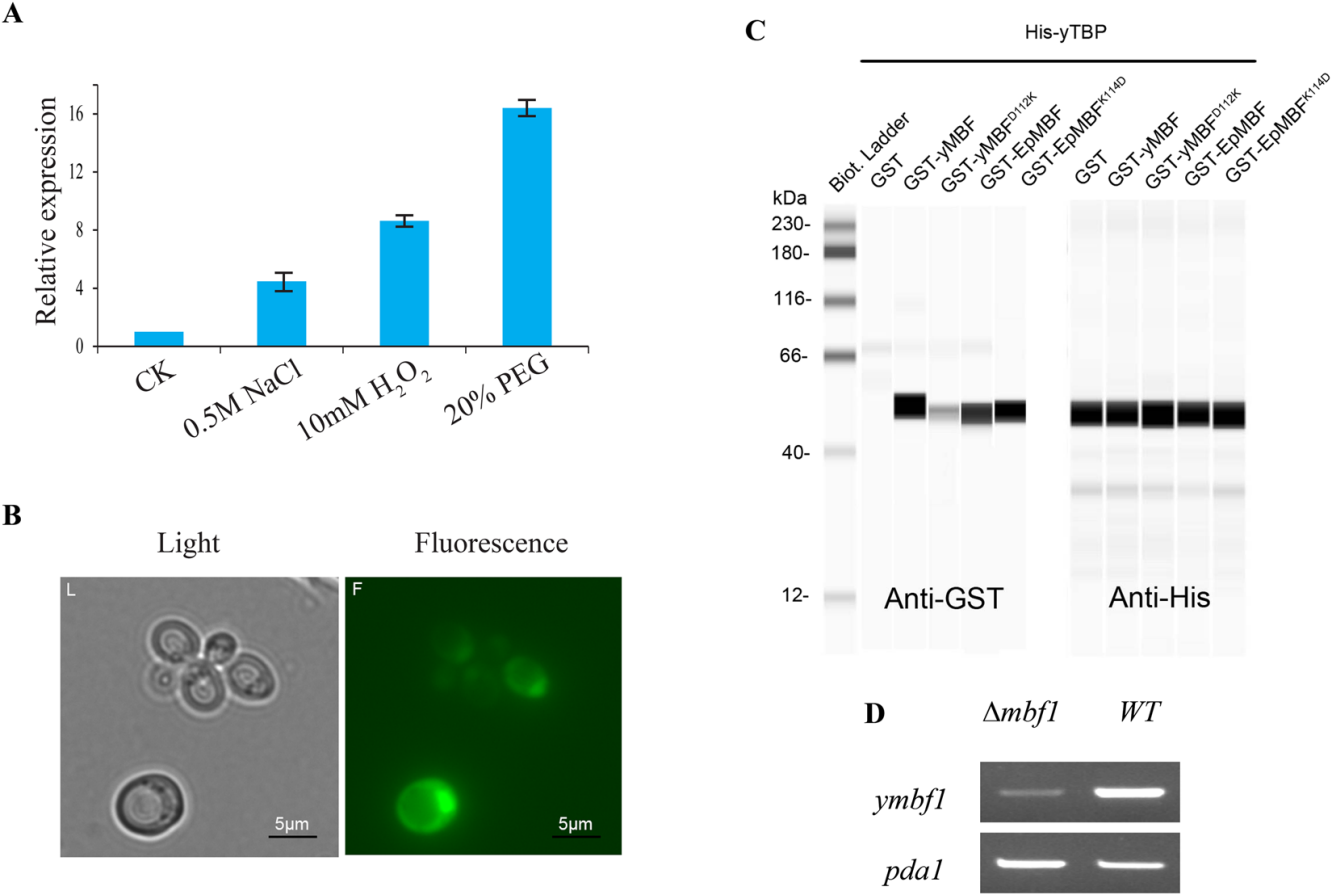
Analysis of EpMBF1 *in vivo* and *in vitro*. (**A**) Expression patterns of *Epmbf1* in *E. pusillum* exposed to different stresses. The abiotic stress treatments were 0.5 mM NaCl, 10 mM H_2_O_2_, and 20% PEG. The expression level in control medium (CK) was considered as the reference. (**B**) Subcellular distribution of the EpMBF1-GFP fusion protein. Scale bar = 5 μm. (**C**) Interactions between yTBP and MBF1s of *E. pusillum* and *S. cerevisiae in vitro* as determined by the GST pull-down assay. (**D**) Semi-quantitative RT-PCR assay to confirm the results of the *ymbf1* gene knock down. *pda1* was used as the reference gene, and its expression level was adjusted and then compared with the expression level of *ymbf1* in the mutant and WT strains. Cropped images are shown; the full length gel and blots are included in Supplementary Information.

To determine whether the expression of *Epmbf1* gene was triggered by stress, the hyphae of *E. pusillum* were exposed to three different stress treatments (high salt, oxidative, and osmotic). As shown in Fig. 2A, after growing on 0.5 mM NaCl-containing medium (salt stress) for 7 d, the *Epmbf1* expression level was up-regulated more than four-fold. Under the 10 mM hydrogen peroxide (H_2_O_2_) treatment (oxidative stress), the *Epmbf1* expression level increased eight times compared with the expression on the control medium. Under the 20% polyethylene glycol (PEG) treatment (osmotic stress), the increased expression level of *Epmbf1* was the highest among those three treatments. Thus, the expression of the *Epmbf1* gene was highly responsive to osmotic stress, moderately responsive to oxidative stress, and slightly responsive to salt stress.

#### EpMBF1 possesses yTBP-*binding* activity and localises to the nucleus

To examine the subcellular localisation of the EpMBF1 protein, an EpMBF1–green fluorescent protein (GFP) fusion gene was introduced into yeast cells. As shown in Fig. 2B, the expressed protein located mainly to the nuclei when the strain was cultured under control conditions. To determine whether a change in the important amino acid K^114^ in the EpMBF1 sequence affected its interaction with yTBP, a glutathione S-transferase (GST) pull-down assay was performed. As shown in Fig. 2C, the original protein EpMBF1^K114^ and point-mutated protein EpMBF1^K114D^ came into direct contact with yTBP, and the blank control protein, GST, did not bind to yTBP. The previous results were also repeated in this experiment, that is, the same amounts of yMBF and yMBF^D112K^ proteins were added into His-yTBP beads, but only yMBF can interact with yTBP.

#### EpMBF1 and yMBF1–EpMBF1 chimeric proteins confer stress resistance in the Δ*ymbf1* mutant

The expression of the *ymbf1* gene in both the Δ*ymbf1* mutant and the wild type (WT) strains was detected using semi-quantitative RT-PCR. The transcription level of the *ymbf1* gene in the mutant strain was significantly lower than in the WT strain, even when a higher concentration of the Δ*ymbf1* strain was used as the template (Fig. 2D). Because the transcriptional level of the *ymbf1* gene was close to zero and the target band of *ymbf1* gene could not be amplified using specific primer pairs (details presented in the Methods section), we considered the gene knock-down strain to be successfully constructed.

**Figure 3:**
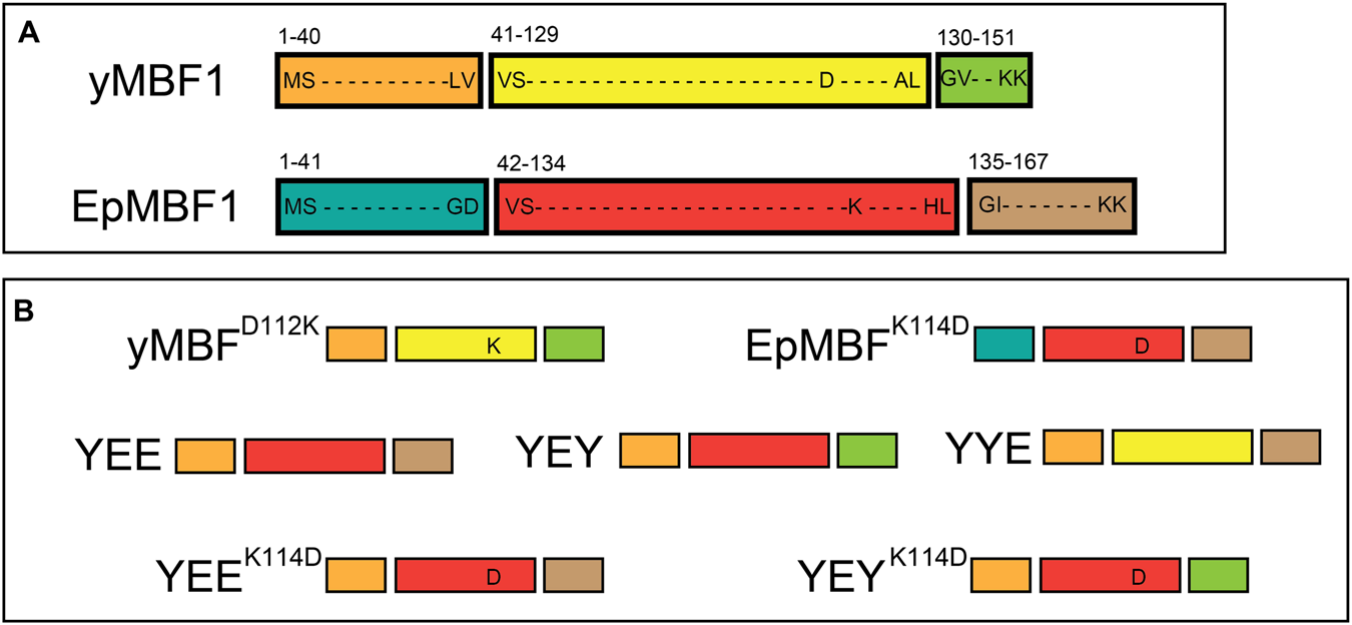
Schematic overview of the EpMBF1–yMBF1 chimeric proteins. (**A**) Original amino acid sequences of yMBF1 and EpMBF1, with different colours indicating the different functional parts. (**B**) Rearranged amino acid sequences used to construct the EpMBF1–yMBF1 chimeric proteins.

**Figure 4:**
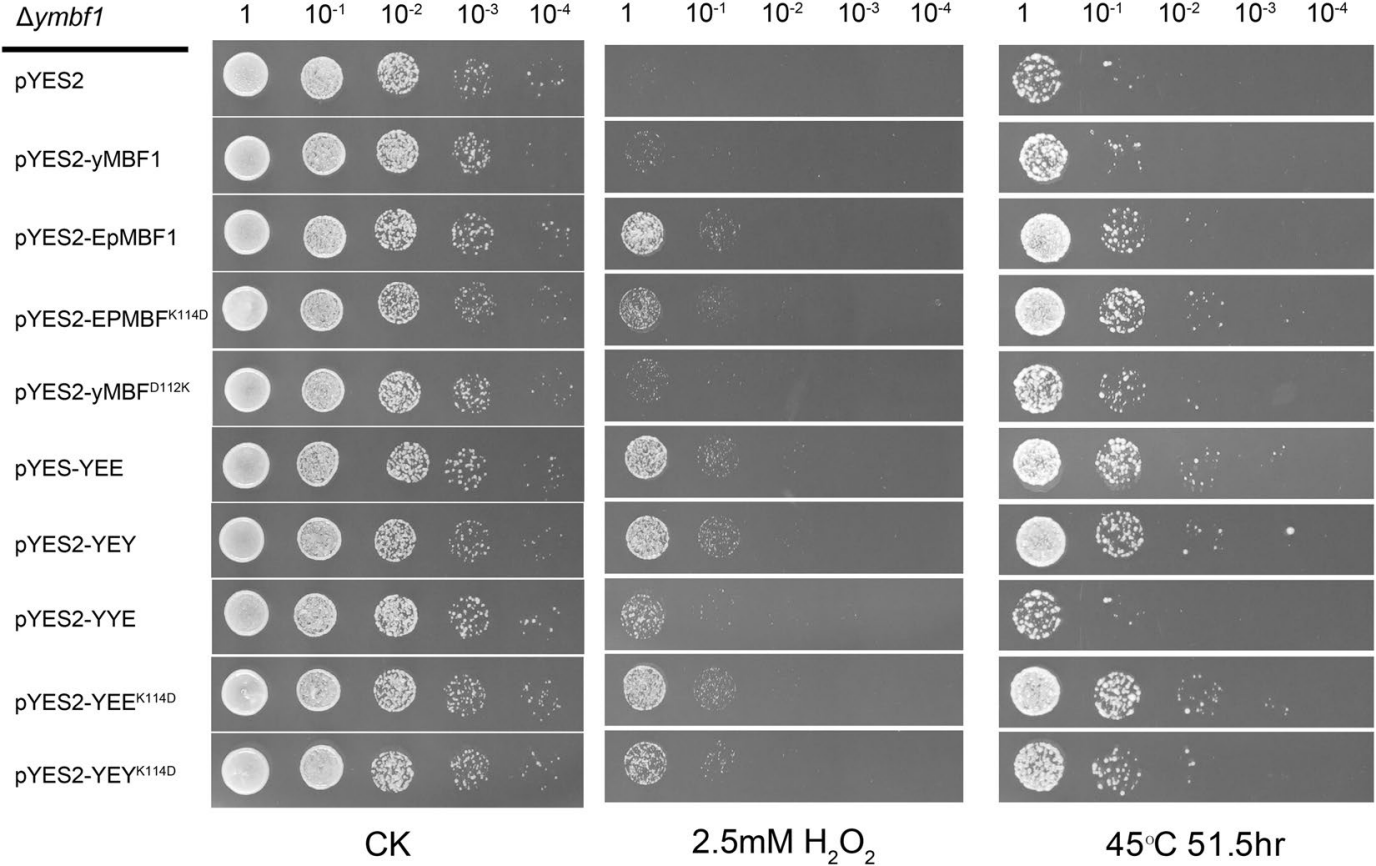
Effects of MBF1s and the chimeric proteins on oxidative and heat stress tolerance in yeast mutants. Yeast strains grown in a concentration grade on selective plates with no treatment (CK), 2.5 mM H_2_O_2_, and 51.5 h at 45 °C, respectively. Photos were taken after 72 h at 30 °C.

To compare the stress-resistant capacities of EpMBF1 and yMBF1, functional complementation assays were performed. And to investigate the potential functions of K^114^ and the extra amino acid region in the EpMBF1 sequences, five chimeric proteins were constructed (Fig. 3) and used, together with two original proteins and two point-mutation proteins, to form the following six comparisons: yMBF1 *vs*. EpMBF1, EpMBF1 *vs*. EpMBF^K114D^, yMBF1 *vs*. yMBF^D112K^, YEE *vs*. YEY, YEE^K114D^ *vs*. YEY^K114D^, and YEE *vs*. YYE. As shown in Fig. 4, the *ymbf1* knock-down strain (Δ*ymbf1–*pYES2) hardly survived and all the transgenic strains were influenced by stress. Comparison of the survival rates of the yMBF1 and EpMBF1 transgenic strains under oxidative and heat stresses, indicated that the EpMBF1 protein improved the stress resistance of the transgenic strain by nearly 10-fold. Comparing EpMBF1 *vs*. EpMBF^K114D^ and yMBF1 *vs*. yMBF^D112K^ showed that the mutant K^114^D in EpMBF1 slightly decreased (less than 10-fold) the oxidative resistance in the transgenic yeast, while the yMBF1 protein did not have this effect. No obvious difference was detected in the stress tolerance of the YEE and YEY transgenic strains, however, the survival rate of the YEY^K114D^ strain was slightly lower than that of the YEE^K114D^ strain under both stresses. Comparison of the survival rates of the YEE and YYE transgenic strains showed that the stress resistant ability of the former protein was better than the latter one, especially under heat stress. From the results of the last two comparisons, it can be concluded that both the C-terminus and the middle region of EpMBF1 contribute to the stress resistant related function. Because the chimeric protein was artificially constructed, we did not compare the original and point mutation-containing proteins in this study.

#### *Epmbf1* mediates the expression patterns of stress-related genes in transgenic yeast

**Figure 5:**
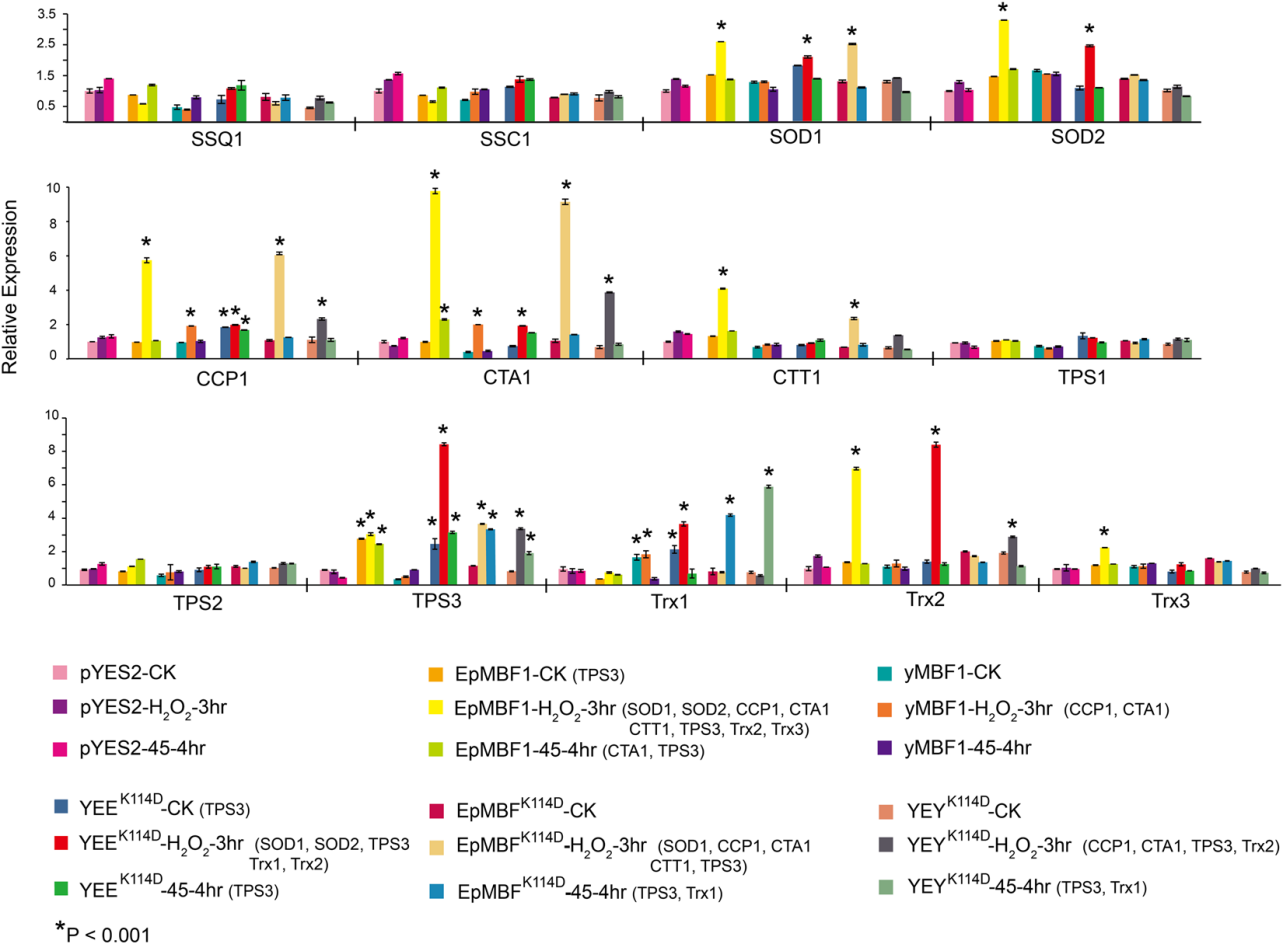
Expression analysis of stress-related genes in transgenic yeast under control and stress conditions. *pda1* was used as the reference gene, and Δ*mbf1/pyes2* was used as the reference sample. Asterisks indicate a significant difference (*P < 0.001) compared with the reference sample (CK). Genes with more than twofold differential expressions are listed with coloured labels.

We performed a RT-PCR assay to determine the consequences of gene expression in transgenic yeast and confirm potential gene targets regulated by *Epmbf1*. Because the mutant K^114^D of YEE and YEY chimeric proteins might participate in protein-protein interaction in yeast cells, and no difference was found between the stress tolerance of YEE and YEY in the functional complementation assay, the differentially expressed genes in these two transgenic strains were not examined. As expected, the oxidative and heat stress treatments did not induce the differential expression of these genes in the Δ*ymbf1–pyes2* transgenic strain (Fig. 5). The over-expression of the *ymbf1* gene in the Δ*ymbf1* mutant up-regulated the expression of the Cytochrome c Peroxidase gene (*ccp1*) and Catalase A gene (*cta1*) under oxidative (H_2_O_2_) stress, indicating that the signal pathway of *ymbf1* may regulate these two genes. In addition, six other downstream genes, SuperOxide Dismutase 1 (*sod1*), SuperOxide Dismutase 2 (*sod2*), Cytosolic catalase 1 (*ctt1*), Trehalose Phosphate Synthase 3 (*tps3*), Thioredoxin 2 (*trx2*), and Thioredoxin 3 (*trx3*), were induced by *Epmbf1* under oxidative stress. Among them, *tps3* was constitutively up-regulated in *Epmbf1* transgenic yeast, because it was significantly differentially expressed under the control conditions. However, only *sod1*, *ccp1*, *cta1*, *ctt1* and *tps3* were differentially expressed in the Δ*ymbf1–Epmbf1* ^*K114D*^ transgenic strain, implying that D at the position 114 influenced the transcriptional regulation of *Epmbf1*. In the Δ*ymbf1–yey* ^*K114D*^ and Δ*ymbf1–yee* ^*K114D*^ strains, the former strain had four up-regulated genes (*ccp1*, *cta1*, *tps3*, and *trx2*) under oxidative stress and two up-regulated genes (*tps3* and Thioredoxin 1 (*trx1*)) under heat stress. The latter had five up-regulated genes (*sod1*, *sod2*, *tps3*, *trx1* and *trx2*) under oxidative stress and one up-regulated gene (*tps3*) under heat stress, among which *sod1*, *tps3*, and *trx1* were constitutively up-regulated. Ruling out the influence of position 114 in yeast cells, this result indicated that the C-terminus of EpMBF1 may induce different downstream genes compared with the corresponding C-terminus region of yMBF1.

## Discussion

For organisms that live in extreme habitats, the adaptability to the environment will be reflected on their genomic information. Identification of genes in lichenized fungi involved in the process of stress tolerance will advance the knowledge of resistant mechanism and also might be beneficial for genetic manipulation of other organisms. Here we identified four unique amino acid sequences that were closely affiliated with the MBF1 protein family in lichens. In contrast to previous results in yeast, we found that the K^114^-specific form, which was previously thought to be functionless in yeast MBF1 protein, had a specific function that was triggered by environmental stresses. Furthermore, the *Endocarpon*-specific C-terminus of the deduced EpMBF1 protein was found to participate in stress tolerance. The known MBF1s, from Archaea, Fungi, Animalia, and Planta, have been classified into three groups^13^. Here, we reported a new MBF1 group from the lichen-forming fungi *Endocarpon* spp. The reconstructed phylogeny (Bayesian tree) based on protein sequences revealed that the newly identified EpMBF1 formed a separate node different from other lichen-forming fungi and Ascomycota in general. Below we discuss the significance of our findings and the possibly evolutionary history of MBF1.

The topologies of both the Bayesian and NJ trees based on MBF1 protein sequences showed that MBF1s in *Endocarpon* spp. formed a separate group. In the NJ tree, the *Endocarpon* group clustered together with the fungi group with a very low bootstrap value which suggest an uncertain phylogenetic situation. However, the well supported Bayesian tree (Fig. 1) showed that this group is different from all the other known MBF1s. Considering the taxa sampling is wide and amino acid evolutionary patterns are distinct, the Bayesian method will be more reliable than the NJ method in this study. Lichen is not a monophyletic group, and all known members were shown to be nested within Fungi^23–26^. The reasons why the Bayesian topology of the MBF1 contradicts the phylogenetic relationships among all the taxa that were included in this study are believed to be the function of the amino acids. Many studies have shown that environmental factors affect the polymorphism of fungal homologous genes^27,28^. And a very recent study on the lichenized fungus *Lasallia pustulata* detected the differentiated genome regions associated with stress response^29^. *Endocarpon* spp. grow in desert areas, therefore, it is assumed that the stress resistance related gene *Epmbf1* was modified and evolved to adapt to this harsh environment.

The MBF1s in this new group had a specific interaction site, K^114^, and an extended sequence at the C-terminus compared with the MBF1s in the other three groups (Supplementary Fig. S1). The amino acid residue at the position 112 in yMBF1, which corresponds to the 114 position of EpMBF1, is important for its interaction and co-evolution with the yTBP site^5^. In this study, we found K, T, and L residues in the corresponding sites of lichen MBF1s (Supplementary Fig. S1), which have not been reported previously in other eukaryotic lineages. Furthermore, we functionally verified the importance of the allele encoding K^114^ in EpMBF1. Previous studies using yeast as a model system indicated that if this interaction site of MBF1 was mutated to K, then the corresponding protein would lose its function^5^. However, based on our GST pull-down and functional assays, we found that the K^114^ site in *E. pusillum* interacted with yTBP and had a strong stress tolerance function (Figs 2C and 4–5). Our results showed that the yeast-based interaction model is not suitable for lichenized fungi. We speculate that the interaction between MBF1 and TBP might not be dependent on one amino acid site. The mutation at the position 112 of yeast MBF1 protein might alter its three-dimensional structure, thus leading to changes on the actual interaction region.

In this study, we found that the main difference between the *Endocarpon* MBF1s and others at the C-terminus is the extra amino acids region (Supplementary Fig. S1). And we showed that the C-terminus of EpMBF1 improved the stress tolerance of transgenic yeast, as indicated by a comparison of the survival rates of the YEY^K114D^ and YEE^K114D^ transgenic strains. Thus, the extended amino acid sequence at the C-terminus of EpMBF1 appears to be responsible for its stress-related functions. Lineage-specific modifications and neo-functionalisation to adapt to distinct niches may explain the functional differences related to the K^114^ (corresponding D^112^) locus between yeast and lichens. For example, *E. pusillum* grows in desert areas and lichens often occur in extreme habitats. MBF1 is related to stress tolerance and this function was validated in our study. The functional locus K^114^ and the extra amino acid fragment at the C-terminus indicated that MBF1s from *Endocarpon* spp. may have evolved a different, yet specific, function in lichens. In addition to lichen, one of the most famous types of resistant fungi is black fungi. Most of the black fungi are affiliated with the Chaetothyriomycetidae, as is *Endocarpon* ^30^. Because the black fungi, like lichenized fungi, live in extreme habitats, grow slowly, and lack genetic tools, their biology is still poorly understood^31^. However, a similar adaptation to extreme environments was found in black yeast. A novel *HAL* gene, which has a special META amino acid region, was isolated from the extremely halotolerant black yeast *Hortaea werneckii* ^32^. In addition, insertion of this specific motif into a plant homologue improves the stress tolerance of plants^33^. Therefore, the extreme adaptive mechanism can be reflected in the functional gene sequence. And the stress related specific motif could function in heterologous organisms.

The expression pattern of *Epmbf1* in *E. pusillum* under these stresses suggests that it participates in regulating the downstream antioxidant genes under oxidative stress. Considering that *E. pusillum* possessed potential stress-tolerant gene resources and the difficulty of direct genetic manipulations in lichen-forming fungi, we performed a heterologous expression analysis in a yeast expression system to study the functional proteins of lichen-forming fungi in order to evaluate their value in improving the stress tolerance of other organisms. After verifying that EpMBF1 was correctly locate in the yeast cells and that it interacted with yTBP (Fig. 2B,C), we performed a functional complement assay. The stress-resistant ability of the EpMBF1 transgenic strain was much greater than that of yMBF1 (Fig. 4). The stress-related phenotypes of these transgenic yeast strains were corroborated by the differential expression of antioxidant genes as determined by RT-PCR (Fig. 5). Thirteen stress-related genes, which were related to resistance to oxidative and heat stresses, were chosen to verify the transcriptional regulation of *Epmbf1* ^34–40^. These 13 candidate downstream genes were selected because they represented the main signal pathways regulated by *mbf1* ^13,17^. As expect, more downstream genes were up-regulated in the *Epmbf1* transgenic strains than in the other strains (Fig. 5). Together, the evidence shows that *Epmbf1* is a biotechnologically promising candidate gene. Further research is required to obtain a more comprehensive understanding of the biological function of EpMBF1.

In conclusion, MBF1s were confirmed in lichens for the first time, and a new MBF1 group was discovered in this gene family in *Endocarpon* spp. Our study shows that the deduced EpMBF1 protein has a strong stress-tolerant ability against abiotic factors.

## Methods

### Strains and cultivation

The lichen-forming fungus, *E. pusillum* (strain Z07020), was isolated from a lichen thallus growing in the Tengger Desert, China. It grew in potato dextrose liquid or potato dextrose agar medium at 18 °C. For the stress media, 0.5 mM NaCl (final concentration), 10 mM H_2_O_2_ (final concentration), or 20% PEG (w/v) were added independently to the potato dextrose agar medium. To simulate drought stress under laboratory conditions, the hyphae of *E. pusillum* were exposed to these media for 7 d at 18 °C.

The WT reference yeast strain was *S. cerevisiae* ‘BY4741’ (*MAT*a, *his3*Δ*1, leu2*Δ0*, met15*Δ0, and *ura3*Δ*0*), which was cultivated at 30 °C in a yeast nitrogen base medium supplemented with 0.5% (w/v) ammonium sulphate, 0.8% (w/v) complete amino acid supplement mixture with or without uracil, and 2% (w/v) glucose, independently. The induction medium contained 2% (w/v) galactose and 1% (w/v) raffinose instead of glucose. The yeast peptone dextrose medium contained 1% yeast extract, 2% peptone, and 2% glucose, with or without 2% agar (all w/v).

### DNA extraction, PCR amplification, and sequencing

DNA was extracted from *E. pusillum* (strain ‘Z07020’) following a modified CTAB method^41^. The primers used for the PCR amplification of the *mbf1* gene were listed in Supplementary Table S1. Reactions were carried out in 50 µl reaction volumes containing 50–1000 ng DNA template in 3 µl stock-solution, 1 µl each primer (10 µM), 25 µl 2× Taq Master Mix (CWBIO, China), and 20 µl ddH_2_O. PCR amplifications were carried out in a Biometra T-Gradient thermal cycler, under the following conditions: initial heating step for 5 min at 95 °C, 35 cycles of 30 s at 94 °C, 30 s at 56 °C, and 1 min at 72 °C, followed by a final extension step of 8 min at 72 °C, after which the samples were kept at 4 °C. Negative controls were prepared for each amplification series. PCR products were cloned and then sequenced by the Shanghai BioSune Corporation of China using an ABI 3730 XL Sequencer. Based on the coding sequence of *Epmbf1*, three *mbf1s* were amplified from *E. crystallinum*, *E. nigromarginatum* and *E. sinense* (GenBank: KX364209, KX364211 and KX364210, respectively) and sequenced.

### Alignment and sequence analysis

Other five MBF1s from the genomes of the lichen-forming fungi, *C. metacorallifera* ^19^, *C. macilenta* ^20^, *G. flavorubescens* ^21^, *U. muehlenbergii* ^22^ and *L. pustulata* (GenBank assembly accession: GCA_900169345.1), were annotated using a local BLASTP algorithm-based search tool. Based on the previous study, 48 MBF1 amino acid sequences were divided into three groups by using NJ tree. Among these sequences, 30 representative sequences were selected used in this study. Including the four *Endocarpon* MBF1 sequences from this study, a dataset of 39 MBF1 sequences were included. These sequences from representative species of other fungi, animals, plants and archaea, in addition to the above mentioned lichen species, were aligned using the MAFFT multiple alignment tool^42^ in BioEdit 7.2.5^43^. The introns were excluded manually and the ambiguously aligned regions were removed by using Gblocks Server (http://molevol.cmima.csic.es/castresana/Gblocks_server.html). The alignment was used to perform NJ and Bayesian Markov chain Monte Carlo analyses with 1,000 pseudo replicates with Mega 5^44^ and MrBayes 3.2.6^45,46^, respectively, on the CIPRES Science Gateway (http://www.phylo.org)^47^. The best model for constructing the Bayesian tree was determined using PhyML^48^ online (http://www.atgc-montpellier.fr/). Two parallel Markov chain Monte Carlo runs were performed each using 8,000,000 generations and sampling every 1,000 steps. A 50% majority rule consensus tree was generated from the sampled trees of both runs after discarding the first 25% as burn-in. The Bayesian results were visualised with FigTree 1.4.2 (http://tree.bio.ed.ac.uk/software/figtree/).

### *Epmbf1* gene expression profiles in *E. pusillum* under abiotic stresses

The total RNA of *E. pusillum* was extracted from each abiotic stress treatment samples using TRIzol reagent (Invitrogen, USA). cDNA syntheses were carried out according to the protocol described in the manual of Reverse Transcriptome System (Promega, USA). Real-Time SYBR Green/ROX PCR master mix (TaKaRa, China) was used for the RT-PCR analysis. The relative quantification of each transcript was calculated by the 2^ΔCT^ method. The translation elongation factor *ef1a* gene was used as an internal control for *E. pusillum*. For each sample, the RT-PCR assay was repeated three times with three biological replicates. Primer pairs are list in Supplementary Table S1.

### Generation of the *S. cerevisiae* Δ*mbf1* mutant strain and semi-quantitative RT-PCR

To investigate the function of EpMBF1 in yeast, we constructed the Δ*mbf1* yeast mutant strain. The *Δymbf1* mutant was constructed using homologous recombination to knock down *ymbf1*. Based on the genomic sequence of *S. cerevisiae*, up- and downstream fragments, ymbf1U (400 bp) and ymbf1D (400 bp), respectively, of the *ymbf1* gene were amplified by PCR, and the KanMX marker for G418 resistance from plasmid pUG6 was cloned. The PCR primers used in this study were listed in Supplementary Table S1. Fragments ymbf1U, KanMX, and ymbf1D were connected through overlapping PCRs, and the recombinant cassette U-KanMX-D was the knock-down component for the *ymbf1* gene. The component was transformed into BY4741a by double-homologous recombination, and then *ymbf1* gene deletion mutants were constructed and selected by the growth extent on yeast peptone dextrose agar plates containing 600 mg/mL G418.

The homologous recombination assay was tested by PCR, and the DNA template of *ymbf1* knock-down strain was examined with the primer pair of ymbf1-F & overlap-ymbf-down-R and overlap-ymbf-up-F & ymbf1-R (Supplementary Table S1). In the Δ*ymbf1* mutant strain, there should not have been any amplification product of *ymbf1* gene. The efficiency of knock-down was confirmed by semi-quantitative RT- PCR^49^. The expressions of the *ymbf1* gene in WT and Δ*ymbf1* mutant strains were compared, and the yeast *pda1* gene was used as the reference gene.

### Subcellular location

To examine the subcellular localisation of EpMBF1 in yeast, the *gfp* gene fragment was amplified by PCR with the primers listed in Supplementary Table S1, and the product was then cloned into pYES2::*Epmbf1*. The EpMBF1–GFP gene encoding the fusion protein was introduced into yeast cells. The fusion protein was expressed in transgenic yeast cells under the control of the galactose promoter, and cells were cultured under control conditions.

### Generation of the point mutation and chimeric proteins

The gene sequences of mutant proteins were constructed by point-mutation-introducing PCR and overlapping PCRs with special primers (Supplementary Table S1). In this study, the K to D mutation at position 114 in EpMBF1 was marked as EpMBF1^K114D^, while the D to K mutation at position 112 in yMBF1 was marked as yMBF1^D112K^.

Because EpMBF1 had a special interaction site in the third helix and extra amino acids at the C-terminus, the main functional region of EpMBF1 was determined by constructing and co-transforming yMBF1–EpMBF1 chimeric proteins and EpMBF1 into the Δ*mbf1* yeast mutant strain together. Five chimeric variants were constructed to determine whether the presence of the extra sequence at the C- terminus of EpMBF1 had any biological function, and whether the major part of the EpMBF1 protein contributed to its activity. Based on its secondary structure, the protein was divided into three parts: the N-terminal domain, amino acids 1 to 40 in yMBF1 and 1 to 41 in EpMBF1; the HTH domain, amino acids 41 to 129 in yMBF1 and 42 to 134 in EpMBF1; and the C-terminal domain, amino acids 130 to 151 in yMBF1 and 135 to 167 in EpMBF1. The chimeric protein YEE contained the N-terminal domain of yMBF1, and the HTH and C-terminal domains of EpMBF1, the chimeric protein YYE contained the N-terminal and the HTH domains of yMBF1, and the C-terminal domain of EpMBF1, while the chimeric protein YEY contained the N-terminal and C-terminal domains of yMBF1, and the HTH domain of EpMBF1. The chimeric protein YEE^K114D^ contained the N-terminal domain of yMBF1, the HTH domain of EpMBF^K114D^, and the C-terminal domain of EpMBF1, and chimeric protein YEY^K114D^ contained the N-terminal and C-terminal domains of yMBF1, and the HTH domain of EpMBF^K114D^. According to previous research, the N-terminus did not contribute much to the activity levels of the MBF1s, and the N-terminal sequence in EpMBF1 was also not special. Therefore, we did not construct the chimeric proteins with N-terminal domains from EpMBF1 in this study. Every part of the MBF1 protein was presented in its original order in the new chimeric proteins.

### Expression of *mbf1* homologs in *S. cerevisiae* and functional complementation assay

To investigate the potential functions of EpMBF1 and its chimeric proteins in yeast, functional complementation assays were carried out. The gene sequences of *mbf1* homologs, mutants, and the chimeric proteins were amplified by PCR with primers listed in Supplementary Table S1. The products were then cloned independently into pYES2, and the final plasmids were confirmed by sequence analyses. Then, the plasmids were transformed into the Δ*ymbf1* mutant. The Δ*mbf1* yeast mutant was transformed with the empty vector (pYES2) as a reference strain to verify that the mutant strain was viable. The Δ*mbf1* mutant carried *ymbf1* in the pYES2 vector as a positive control for survival rate comparisons with other transgenic yeast strains under oxidative and heat stresses. For the oxidative stress, the strains were cultured on medium containing 2.5 mM H_2_O_2_, and the culture plates were examined after 3d. For the heat stress treatment, the strains were incubated at 45 °C for 51.5 h and then recovered at the control temperature (30 °C) for 3d.

### The GST pull-down assay

The GST pull-down assay was used to examine the binding of yTBP to EpMBF1 and EpMBF^K114D^ *in vitro*. The coding sequence of each tested gene was amplified from the cDNA sequence. The cDNA fragment of yTBP was inserted into pET-32a vector to link the His tag, while the cDNA fragments of yMBF1, yMBF^D112K^, EpMBF1, and EpMBF^K114D^ were cloned into pGEX6p-1 vector to link the GST tag. The final plasmids were transformed into *Escherichia coli* strain BL21. Soluble proteins (the supernatant of cell lysate) with GST-tag and His-tag were incubated with 250 μl glutathione agarose beads and Ni-NTA agarose beads (Invitrogen) for 4 h at 4 °C, respectively. The beads were washed three times, and the target proteins were eluted using elution buffer. The purified proteins were detected by electrophoresis (Supplementary Fig. S3), and concentrations of these proteins were determinate using Qubit 2.0 (Invitrogen, USA). Five equal amounts of soluble proteins with His-tag were incubated with 250 μl Ni-NTA agarose beads at 4 °C for 4 h, and then the beads were washed three times. The same amount of four purified MBF1 proteins and the purified GST protein incubated with these five His-yTBP columns for another 4 h at 4 °C. The beads were washed repeatedly three times, and the presence of pull-down proteins was detected by immunoblot using anti-His and anti-GST antibodies. The western blot assay was performed using Wes^TM^ (ProteinSimple, USA).

### The stress-related genes expression profiles in transgenic yeast under abiotic stresses

Δ*mbf1/pyes2*, Δ*mbf1/Epmbf1*, Δ*mbf1/ymbf1*, Δ*mbf1/Epmbf1* ^*K114D*^, Δ*mbf1/yee* ^*K114D*^, and Δ*mbf1/yey* ^*K114D*^ transformed strains were used as test samples. The strains were treated with 2.5 mM H_2_O_2_ and at 45 °C for 3 h and 4 h, respectively. Total RNAs were isolated from *S. cerevisiae* cells cultured in liquid medium, and used for cDNA synthesis. The pyruvate dehydrogenase *pda1* gene was used as internal controls for *S. cerevisiae*. For each gene, the RT-PCR assay was repeated three times with three biological replicates. Primer pairs are listed in Supplementary Table S1. The expression levels of the tested genes in the transformant Δ*mbf1/pyes2* in the control medium were considered as reference data. Compared with the reference data, genes with relative expression increases of at least twofold and P values less than 0.001 were considered as differentially expressed genes.

## Electronic supplementary material

Supplementary figures

Dataset 1

Dataset 2

## References

[1] Millership JJ, Waghela P, Cai XM, Cockerham A, Zhu G (2004). Differential expression and interaction of transcription co-activator MBF1 with TATA-binding protein (TBP) in the apicomplexan Cryptosporidium parvum. Microbiol-Sgm.

[2] de Koning B, Blombach F, Wu H, Brouns SJ, van der Oost J (2009). Role of multiprotein bridging factor 1 in archaea: bridging the domains?. Biochem Soc Trans.

[3] Ozaki J (1999). Identification of the core domain and the secondary structure of the transcriptional coactivator MBF1. Genes Cells.

[4] Mishima M (1999). Resonance assignments, secondary structure and 15N relaxation data of the human transcriptional coactivator hMBF1 (57–148). J Biomol NMR.

[5] Liu QX, Nakashima-Kamimura N, Ikeo K, Hirose S, Gojobori T (2007). Compensatory change of interacting amino acids in the coevolution of transcriptional coactivator MBF1 and TATA-box-binding protein. Molecular biology and evolution.

[6] Hedges SB (2002). The origin and evolution of model organisms. Nat Rev Genet.

[7] Abrahamsen MS (2004). Complete genome sequence of the apicomplexan, Cryptosporidium parvum. Science.

[8] Takemaru K, Harashima S, Ueda H, Hirose S (1998). Yeast coactivator MBF1 mediates GCN4-dependent transcriptional activation. Mol Cell Biol.

[9] Kim MJ (2007). Abiotic and biotic stress tolerance in Arabidopsis overexpressing the Multiprotein bridging factor 1a (MBF1a) transcriptional coactivator gene. Biochem Bioph Res Co.

[10] Suzuki N, Bajad S, Shuman J, Shulaev V, Mittler R (2008). The transcriptional co-activator MBF1c is a key regulator of thermotolerance in Arabidopsis thaliana. J Biol Chem.

[11] Tojo T (2009). Arabidopsis MBF1s Control Leaf Cell Cycle and its Expansion. Plant Cell Physiol.

[12] Takemaru K, Li FQ, Ueda H, Hirose S (1997). Multiprotein bridging factor 1 (MBF1) is an evolutionarily conserved transcriptional coactivator that connects a regulatory factor and TATA element-binding protein. P Natl Acad Sci USA.

[13] Ying SH, Ji XP, Wang XX, Feng MG, Keyhani NO (2014). The transcriptional co-activator multiprotein bridging factor 1 from the fungal insect pathogen, Beauveria bassiana, mediates regulation of hyphal morphogenesis, stress tolerance and virulence. Environmental microbiology.

[14] Coto, J. M., Ehrenhofer-Murray, A. E., Pons, T. & Siebers, B. Functional analysis of archaeal MBF1 by complementation studies in yeast. *Biol Direct* 6, 10.1186/1745-6150-6-18 (2011). 10.1186/1745-6150-6-18 PMC3062615 21392374

[15] Tsuda K, Tsuji T, Hirose S, Yamazaki K (2004). Three Arabidopsis MBF1 homologs with distinct expression profiles play roles as transcriptional co-activators. Plant Cell Physiol.

[16] Aravind L, Koonin EV (1999). DNA-binding proteins and evolution of transcription regulation in the archaea. Nucleic Acids Research.

[17] Qin D (2015). Overexpression of heat stress-responsive TaMBF1c, a wheat (Triticum aestivum L.) Multiprotein Bridging Factor, confers heat tolerance in both yeast and rice. Plant molecular biology.

[18] Zhang T, Wei J (2011). Survival analyses of symbionts isolated from Endocarpon pusillum Hedwig to desiccation and starvation stress. Sci China Life Sci.

[19] Park, S. Y. *et al*. Draft Genome Sequence of Lichen-Forming Fungus Cladonia metacorallifera Strain KoLRI002260. *Genome Announc* **2**, 10.1128/genomeA.01065-13 (2014). 10.1128/genomeA.01065-13 PMC3924382 24526650

[20] Park, S. Y. *et al*. Draft Genome Sequence of Cladonia macilenta KoLRI003786, a Lichen-Forming Fungus Producing Biruloquinone. *Genome Announc* **1**, 10.1128/genomeA.00695-13 (2013). 10.1128/genomeA.00695-13 PMC3764418 24009123

[21] Park, S. Y. *et al*. Draft Genome Sequence of Lichen-Forming Fungus Caloplaca flavorubescens Strain KoLRI002931. *Genome Announc* **1**, 10.1128/genomeA.00678-13 (2013). 10.1128/genomeA.00678-13 PMC3757454 23990579

[22] Park, S. Y. *et al*. Draft Genome Sequence of Umbilicaria muehlenbergii KoLRILF000956, a Lichen-Forming Fungus Amenable to Genetic Manipulation. *Genome Announc* **2**, 10.1128/genomeA.00357-14 (2014). 10.1128/genomeA.00357-14 PMC3999499 24762942

[23] Lutzoni F, Pagel M, Reeb V (2001). Major fungal lineages are derived from lichen symbiotic ancestors. Nature.

[24] Lutzoni F (2004). Assembling the fungal tree of life: Progress, classification and evolution of subcellular traits. Am J Bot.

[25] Prieto M, Wedin M (2013). Dating the diversification of the major lineages of Ascomycota (Fungi). PLoS ONE.

[26] Schoch CL (2009). The Ascomycota Tree of Life: A Phylum-wide Phylogeny Clarifies the Origin and Evolution of Fundamental Reproductive and Ecological Traits. Syst Biol.

[27] Branco S (2015). Genetic isolation between two recently diverged populations of a symbiotic fungus. Molecular ecology.

[28] Aguileta G, Refregier G, Yockteng R, Fournier E, Giraud T (2009). Rapidly evolving genes in pathogens: methods for detecting positive selection and examples among fungi, bacteria, viruses and protists. Infection, Genetics and Evolution.

[29] Dal Grande F (2017). Adaptive differentiation coincides with local bioclimatic conditions along an elevational cline in populations of a lichen-forming fungus. BMC Evol Biol.

[30] Gueidan C, Ruibal C, de Hoog GS, Schneider H (2011). Rock-inhabiting fungi originated during periods of dry climate in the late Devonian and middle Triassic. Fungal biology.

[31] Grube, M., Muggia, L. & Gostinčar, C. in *Polyextremophiles* 551–566 (Springer, 2013).

[32] Vaupotič T, Gunde-Cimerman N, Plemenitaš A (2007). Novel 3′-phosphoadenosine-5′-phosphatases from extremely halotolerant Hortaea werneckii reveal insight into molecular determinants of salt tolerance of black yeasts. Fungal Genetics and Biology.

[33] Gašparič MB (2013). Insertion of a Specific Fungal 3′-phosphoadenosine-5′-phosphatase Motif into a Plant Homologue Improves Halotolerance and Drought Tolerance of Plants. PloS one.

[34] Charizanis C, Juhnke H, Krems B, Entian KD (1999). The mitochondrial cytochrome c peroxidase Ccp1 of Saccharomyces cerevisiae is involved in conveying an oxidative stress signal to the transcription factor Pos9 (Skn7). Mol Gen Genet.

[35] Costa V, Moradas-Ferreira P (2001). Oxidative stress and signal transduction in Saccharomyces cerevisiae: insights into ageing, apoptosis and diseases. Mol Aspects Med.

[36] Pujol-Carrion N, de la Torre-Ruiz MAG (2010). Grx4 and Grx3 of Saccharomyces cerevisiae Play a Role in Actin Dynamics through Their Trx Domains, Which Contributes to Oxidative Stress Resistance. Applied and environmental microbiology.

[37] Kamo K, Takabatake A, Inoue Y, Izawa S (2012). Temperature dependent N-glycosylation of plasma membrane heat shock protein Hsp30p in Saccharomyces cerevisiae. Biochem Biophys Res Commun.

[38] Kim IS, Kim YS, Kim H, Jin I, Yoon HS (2013). Saccharomyces cerevisiae KNU5377 stress response during high-temperature ethanol fermentation. Mol Cells.

[39] Martins D, English AM (2014). Catalase activity is stimulated by H2O2 in rich culture medium and is required for H2O2 resistance and adaptation in yeast. Redox Biol.

[40] Trevisol ETV, Panek AD, De Mesquita JF, Eleutherio ECA (2014). Regulation of the yeast trehalose-synthase complex by cyclic AMP-dependent phosphorylation. Bba-Gen Subjects.

[41] Rogers, S. O. & Bendich, A. J. in *Plant molecular biology manual* 73-83 (Springer, 1989).

[42] Katoh K, Misawa K, Kuma K, Miyata T (2002). MAFFT: a novel method for rapid multiple sequence alignment based on fast Fourier transform. Nucleic Acids Res.

[43] Del-Prado R (2010). Genetic distances within and among species in monophyletic lineages of Parmeliaceae (Ascomycota) as a tool for taxon delimitation. Mol Phylogenet Evol.

[44] Tamura K (2011). MEGA5: Molecular Evolutionary Genetics Analysis Using Maximum Likelihood, Evolutionary Distance, and Maximum Parsimony Methods. Molecular biology and evolution.

[45] Huelsenbeck JP, Ronquist F (2001). MRBAYES: Bayesian inference of phylogenetic trees. Bioinformatics.

[46] Ronquist F, Huelsenbeck JP (2003). MrBayes 3: Bayesian phylogenetic inference under mixed models. Bioinformatics.

[47] Miller, M. A., Pfeiffer, W. & Schwartz, T. In *Gateway Computing Environments Workshop (GCE)*, *2010*. 1–8 (IEEE).

[48] Guindon S, Gascuel O (2003). A simple, fast, and accurate algorithm to estimate large phylogenies by maximum likelihood. Syst Biol.

[49] Chen L, Segal DM, Mash DC (1999). Semi-quantitative reverse-transcriptase polymerase chain reaction: an approach for the measurement of target gene expression in human brain. Brain Res Brain Res Protoc.

