# Supplementary material for: Modification and functional adaptation of the MBF1 gene family in the lichenized fungus Endocarpon pusillum under environmental stress
Source: Sci Rep. 2017 Nov 27;7:16333. doi: 10.1038/s41598-017-16716-4 (PMC5703946; doi:10.1038/s41598-017-16716-4)

Modification and functional adaptation of the MBF1 gene family in the lichenized fungus *Endocarpon pusillum* under environmental stress

Yanyan Wang[1], Xinli Wei[1*], Jenpan Huang[2], and Jiangchun Wei[1, 3*]

[1]State Key Laboratory of Mycology, Institute of Microbiology, Chinese Academy of Sciences, Beijing, 10010, PR China

[2]Science & Education, The Field Museum, Chicago, IL 60605, USA

[3]University of Chinese Academy of Sciences, Beijing, 100049, PR China

*corresponding author: weixl@im.ac.cn; weijc2004@126.com

Figure S1. Multiple sequence alignment of MBF1s of fungi from Saccharomycotina and Pezizomycotina in Ascomycota and Agaricomycotina in Basidiomycota. These sequences were aligned using MAFFT. The secondary structures of the MBF1s were predicted using NCBI BLASTP and an online software ESPript 3.033 based on the nuclear magnetic resonance structural information of *Trichoderma reesei* from the Protein Data Bank (http://www.rcsb.org/pdb/explore/explore.do?structureId=2JVL). The red box with white characters indicates strict identity, the red characters indicate similarity in a group, and the blue frame indicates similarity across groups.

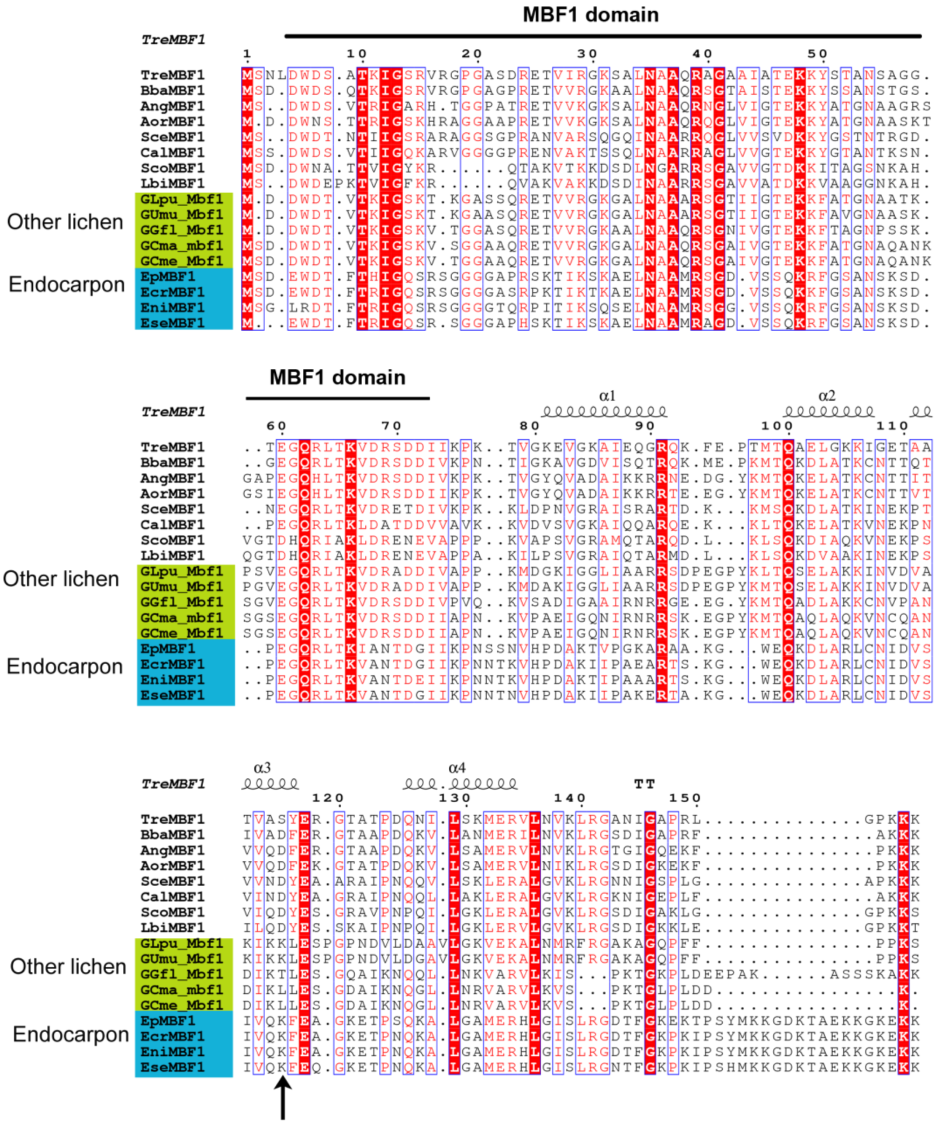

Figure S2. The NJ phylogenetic trees based on the amino acid sequences of MBF1s. Phylogenetic tree was constructed with MEGA version 5.01 by the neighbor-joining method using the Poisson model. In total, 39 MBF1 references representing Archaea, Fungi, Animalia and Planta were selected (Table S2).

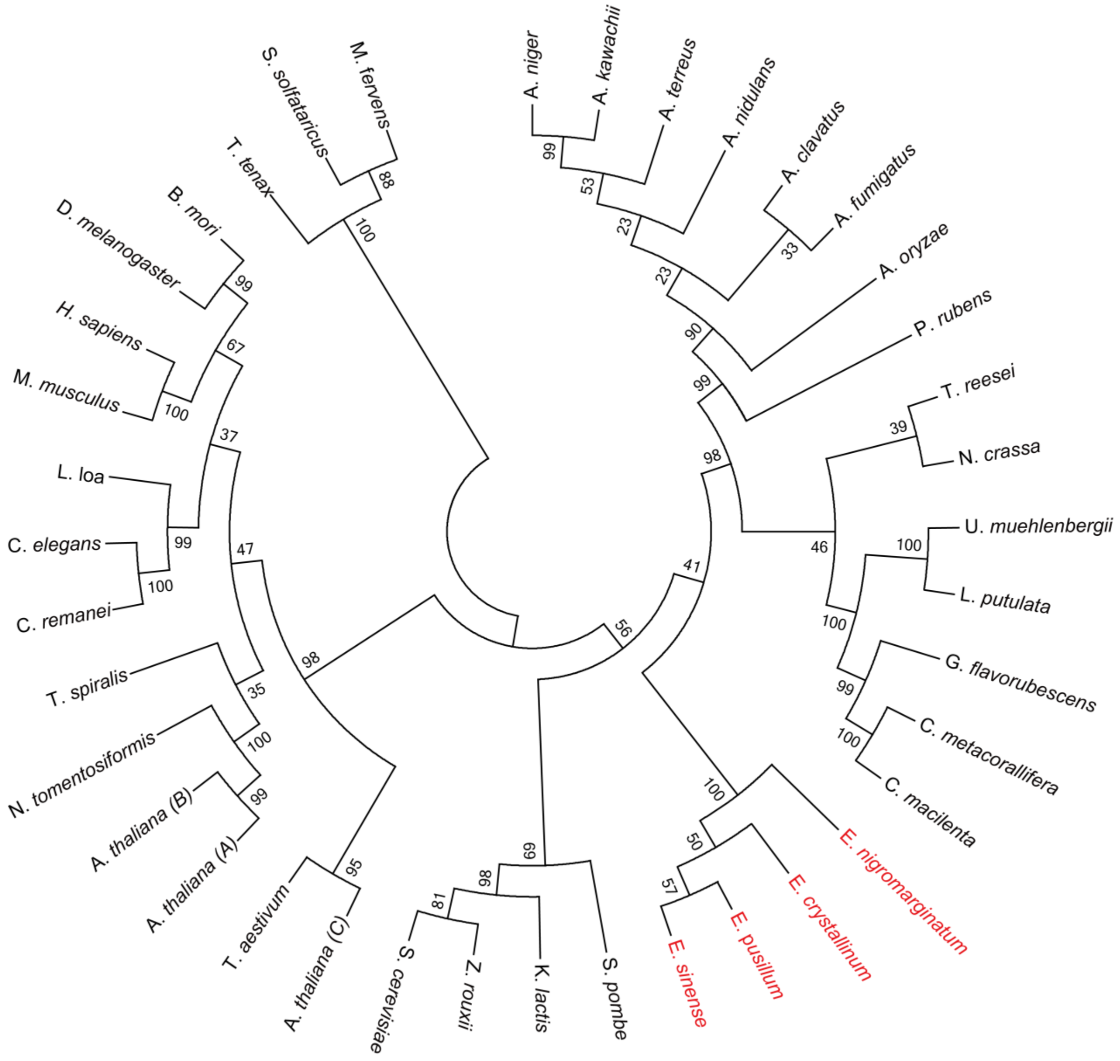

Figure S3. The purified in put proteins for GST pull down assay.

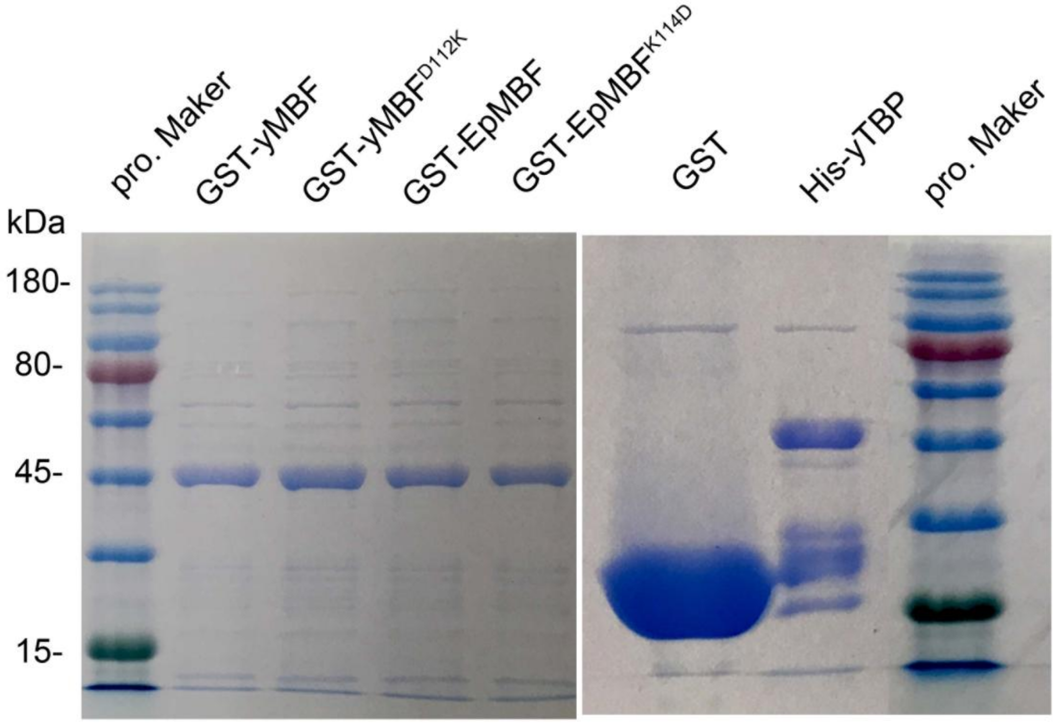

Supplement: Supplementary file 1 — Supplementary figures [file 41598_2017_16716_MOESM1_ESM.pdf]
